# Augmented Lung Inflammation Protects against Influenza A Pneumonia

**DOI:** 10.1371/journal.pone.0004176

**Published:** 2009-01-12

**Authors:** Michael J. Tuvim, Scott E. Evans, Cecilia G. Clement, Burton F. Dickey, Brian E. Gilbert

**Affiliations:** 1 Department of Pulmonary Medicine, The University of Texas M. D. Anderson Cancer Center, Houston, Texas, United States of America; 2 Center for Lung Inflammation and Infection, Institute of Biosciences and Technology, Texas A&M Health Science Center, Houston, Texas, United States of America; 3 Department of Molecular Virology and Microbiology, Baylor College of Medicine, Houston, Texas, United States of America; Institut Pasteur, France

## Abstract

**Background:**

Influenza pneumonia causes high mortality every year, and pandemic episodes kill millions of people. Influenza-related mortality has been variously ascribed to an ineffective host response that fails to limit viral replication, an excessive host inflammatory response that results in lung injury and impairment of gas exchange, or to bacterial superinfection. We sought to determine whether lung inflammation promoted or impaired host survival in influenza pneumonia.

**Methods and Findings:**

To distinguish among these possible causes of influenza-related death, we induced robust lung inflammation by exposing mice to an aerosolized bacterial lysate prior to challenge with live virus. The treatment induced expression of the inflammatory cytokines IL-6 and TNF in bronchoalveolar lavage fluid 8- and 40-fold greater, respectively, than that caused by lethal influenza infection. Yet, this augmented inflammation was associated with striking resistance to host mortality (0% vs 90% survival, p = 0.0001) and reduced viral titers (p = 0.004). Bacterial superinfection of virus infected lungs was not observed. When mice were repeatedly exposed to the bacterial lysate, as would be clinically desirable during an influenza epidemic, there was no tachyphylaxis of the induced viral resistance. When the bacterial lysate was administered after the viral challenge, there was still some mortality benefit, and when ribavirin was added to the aerosolized bacterial lysate, host survival was synergistically improved (0% vs 93.3% survival, p<0.0001).

**Conclusions:**

Together, these data indicate that innate immune resistance to influenza can be effectively stimulated, and suggest that ineffective rather than excessive inflammation is the major cause of mortality in influenza pneumonia.

## Introduction

The annual worldwide mortality associated with pneumonia exceeds that of any other infection [1], [2], [3]. In particular, influenza pneumonia annually causes more than 40,000 deaths in the United States alone [4], [5]. Beyond the impact of seasonal influenza, episodes of pandemic influenza have accounted for as many as 50 million deaths [6]. H5N1 avian influenza has already caused more than 240 human deaths worldwide (http://www.who.int/csr/disease/avian_influenza/), and increased globalization since 1918 suggests that eventual human-to-human transmission of avian influenza may cause even greater lethality than the infamous “Spanish flu” [7]. Further, viral pathogens, including influenza, are considered potential agents of bioterror [8].

The mechanisms underlying influenza-related mortality remain controversial. Progressive pneumonia following an insufficient antiviral host response is one possible cause [9]. Virally-induced excessive and/or dysregulated lung inflammation is another potential mechanism [10], [11], [12], [13]. Secondary bacterial infections have also been proposed as the primary contributors to influenza-related mortality, due to virally-injured epithelium or virus-attenuated leukocyte responses [13], [14], [15].

We have recently reported that treatment with an aerosolized lysate of nontypeable *Haemophilus influenzae* (NTHi) induces profound inflammation in the lungs, yet it strongly protects mice against otherwise fatal bacterial pneumonia [16]. The induced protective phenomenon, known as Stimulated Innate Resistance (StIR), is maximal within 4 h of treatment and does not rely on recruited neutrophils or resident mast cells and alveolar macrophages. Given the profound induction of lung inflammation by this treatment, we perceived an opportunity to determine whether host inflammation contributed to or prevented mortality in influenza A pneumonia.

We demonstrate that, despite inducing inflammation greater than that observed in lethally infected animals, the aerosolized NTHi lysate results in remarkable protection against otherwise lethal influenza pneumonia, and that it can be synergistically combined with antiviral medicine for post-infectious treatment. Together, these data suggest that ineffective rather than excessive inflammation is the major cause of mortality in influenza pneumonia, and indicate that innate immune resistance to viruses can be therapeutically stimulated to protect populations from pandemic influenza.

## Results

### Protection against influenza A pneumonia

**Figure 1:**
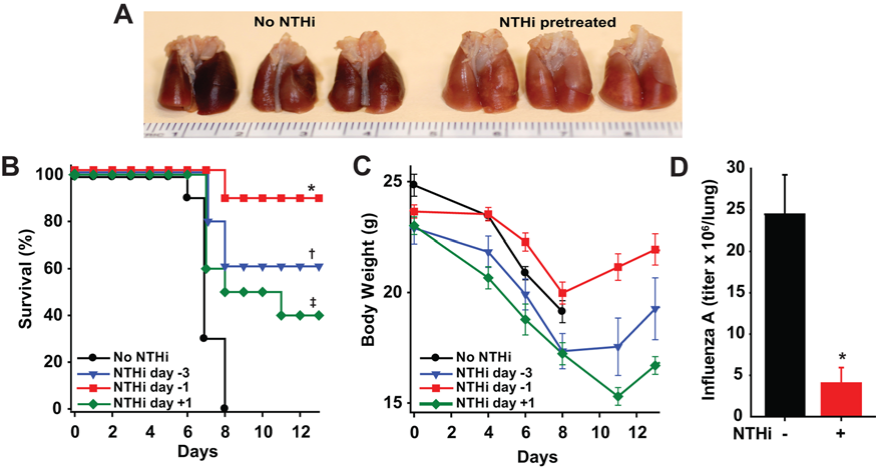
Aerosolized NTHi lysate protects against hemorrhagic influenza virus pneumonia. (A) Gross pathologic images of lungs 7 days after influenza A challenge. (B) Mice were challenged with aerosolized influenza A (2.8×10^4^ TCID_50_/ml) after receiving no NTHi lysate treatment, or NTHi lysate treatment 3 days before, 1 day before, or 1 day after infection (* p = 0.0001, † p = 0.011, ‡ p = 0.043 when each treated group was compared to the untreated group). (C) Weight changes in the same groups of mice shown in A. (D) Viral titers in lung homogenates were measured 4 days after viral challenge, with or without a single NTHi lysate pretreatment 1 day prior to infection (* p = 0.004).

Having shown that profound inflammatory lung responses to a bacterial lysate improved survival of mice infected with non-cognate bacteria [16], we investigated the effect of stimulation of lung mucosal innate immunity on survival of influenza A pneumonia. Mice challenged by aerosol with influenza A/Hong Kong/8/68 (H3N2) (A/HK) universally succumbed to hemorrhagic pneumonia unless pretreated with aerosolized NTHi lysate (Fig. 1A). This treatment reduced mortality >90% if delivered on the day prior to infection, >50% if delivered 3 days prior to infection, and to a lesser degree if delivered one day after infection (Fig. 1B). Mortality occurred within 10 days of the viral inoculation, and observation for 21 days after infection showed no subsequent mortality. The protection imparted by the aerosolized lysate was paralleled by the recovery of body weight lost early in the course of infection (Fig. 1C). Lysate-induced survival was accompanied by a significant reduction in viral load on the fourth day after challenge (Fig. 1D).

### Protection is associated with lung-restricted inflammation

**Figure 2:**
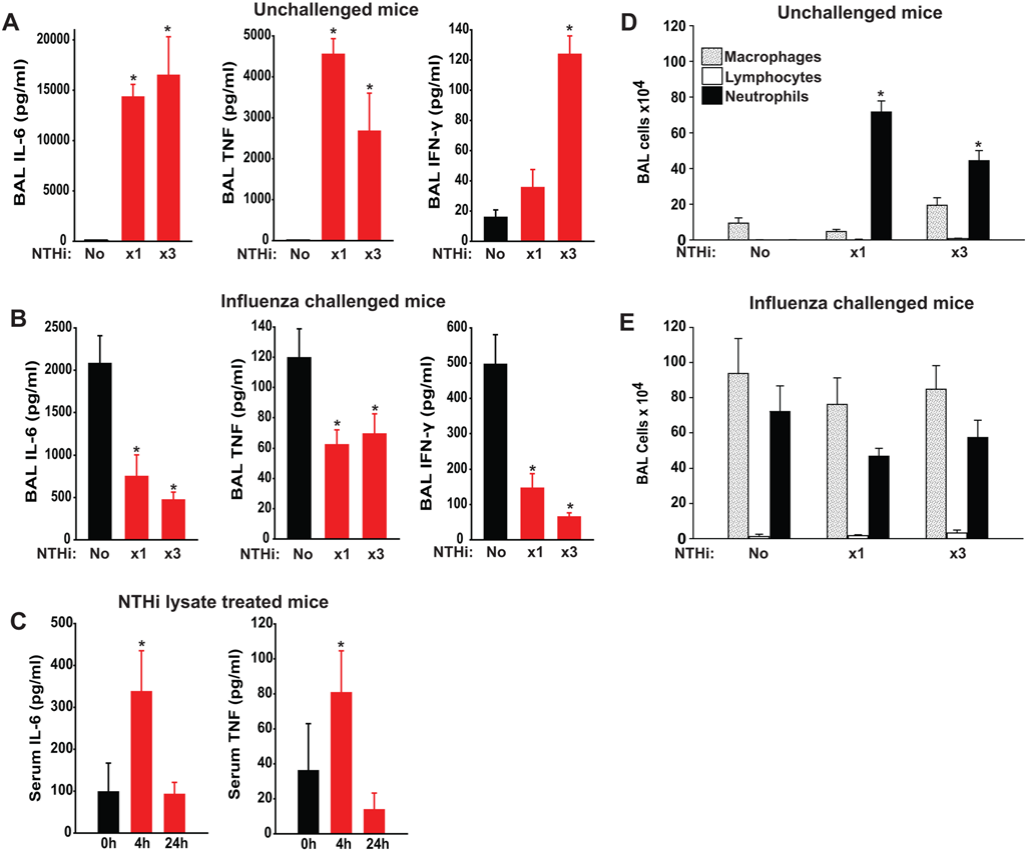
Protection against influenza A is associated with lung-restricted inflammation. (A) Lung inflammatory cytokine levels were determined in mice treated with NTHi lysate once (“×1”), every third day for 6 days (“×3”), or not at all. ELISA was performed on BAL fluid obtained 4 h after the final treatment. (B) Lung inflammatory cytokines were measured 3 days after influenza A infection without NTHi lysate pretreatment, following a single treatment on day −1 before infection, or following treatments on days −7, −4, and −1 prior to infection. (C) Serum levels of inflammatory cytokines at designated timepoints after NTHi lysate treatment. (D) Inflammatory cell counts in BAL fluid of mice treated (or not) with NTHi lysate, as in A. (E) Inflammatory cell counts in BAL fluid of mice treated (or not) with NTHi lysate then infected with influenza A, as in B. (*p<0.01 compared with untreated).

Since prior reports have described poorer outcomes associated with virus-induced inflammation [10], [11], we compared the inflammation induced by the NTHi lysate, inflammation induced by influenza infection, and inflammation induced by the combination of treatment and infection. Figure 2 shows that single or repeated treatment with NTHi lysate induces dramatic inflammation in the lungs, measured by cytokine levels (Fig. 2A) and cellular influx (Fig. 2D).

Remarkably, while treatment with NTHi lysate acutely induces 8-fold more IL-6 and almost 40-fold more TNF than infection alone, the treated mice actually have lower levels of inflammatory cytokines in their lungs by day 3 after the infection (Fig. 2B). By that time after treatment, the NTHi lysate-induced rise in cytokines in uninfected mice has entirely resolved (data not shown), and the inflammatory cell infiltration has fallen by more than 80%[16]. Together, this suggests that a robust early inflammatory reaction may allow for more rapid resolution of the infection-induced inflammation. Notably, the intense inflammation observed in the lungs is not seen systemically. Despite increases in IL-6 and TNF of 700- and 900-fold in the lungs, respectively, these cytokines increase only minimally and transiently in the serum (Fig. 2C).

### Protection is associated with interferon signaling

Since interferon signaling is essential to baseline host antiviral resistance in the lungs [17], [18], we investigated whether treatment with NTHi lysate was capable of inducing an interferon response. In addition to the robust induction of inflammatory cytokines, we found that NTHi lysate treatment also induces significant increases in lung interferon γ levels (Fig. 2A). However, unlike IL-6 and TNF, the fulminant influenza infections observed in untreated mice actually induce 4-fold higher levels of interferon γ than the NTHi lysate treatment (Fig. 2B). Presumably this reflects the ongoing antiviral response of the host, in contrast to the better contained infections of the treated mice.

To further characterize the lung interferon response to the NTHi lysate, we assessed gene expression using whole genome microarray analysis. As maximal resistance against *S. pneumoniae* is achieved by 4 h post-treatment, we compared gene expression of sham treated mouse lungs to mouse lungs 2 and 4 h post-treatment. Pathway analysis revealed interferon signaling to be among the most upregulated events following NTHi lysate treatment (p<10^−11^). Interferon signaling pathway transcripts are reported in Table 1, showing that treatment induces expression of numerous transcripts critical for both type I (interferon-α/β) and type II (interferon-γ) signaling.

**Table 1:** Interferon gene expression changes following NTHi lysate treatment.

Symbol	Definition	2 h fold change	4 h fold change	Accession
*G1p2*	Interferon alpha-inducible protein	4.51	8.00	NM_015783.1
*Icsbp1*	Interferon consensus sequence binding protein 1	1.20	1.07	NM_008320.2
*Ifi1*	Interferon inducible protein 1	3.68	2.38	NM_008326.1
*Ifi16*	Interferon gamma-inducible protein 16	1.22	2.24	NM_008329.1
*Ifi202b*	Interferon activated gene 202B	2.48	ND	NM_008327.1
*Ifi203*	Interferon activated gene 203	1.44	2.40	NM_008328.1
*Ifi205*	Interferon activated gene 205	24.22	53.00	NM_172648.2
*Ifi30*	Interferon gamma inducible protein 30	1.044	0.72	NM_023065.2
*Ifi35*	Interferon-induced protein 35	1.20	1.34	NM_027320.1
*Ifi47*	Interferon gamma inducible protein	4.66	3.60	NM_008330.1
*Ifit1*	Interferon-induced protein with tetratricopeptide repeats 1	ND	ND	NM_008331
*Ifit2*	Interferon-induced protein with tetratricopeptide repeats 2	2.01	4.23	NM_008332.2
*Ifit3*	Interferon-induced protein with tetratricopeptide repeats 3	3.08	4.21	NM_010501.1
*Ifitm1*	Interferon-induced transmembrane protein 1	2.66	2.59	NM_026820.2
*Ifitm2*	Interferon-induced transmembrane protein 2	1.75	1.36	NM_030694
*Ifitm3*	Interferon-induced transmembrane protein 3	1.70	1.34	NM_025378.1
*Ifitm5*	Interferon-induced induced transmembrane protein 5	1.53	2.40	NM_053088
*Ifitm7*	Interferon-induced transmembrane protein 7	0.70	0.72	NM_028968
*Ifna1*	Interferon alpha family gene 1	1.74	1.69	NM_010502.1
*Ifna2*	Interferon alpha family gene 2	ND	ND	NM_010503.1
*Ifna4*	Interferon alpha family gene 4	ND	ND	NM_010504.1
*Ifna5*	Interferon alpha family gene 5	0.93	0.81	NM_010505
*Ifna6*	Interferon alpha family gene 6	ND	9.08	NM_008335.1
*Ifna7*	Interferon alpha family gene 7	1.50	0.55	NM_008334
*Ifna9*	Interferon alpha family gene 9	ND	ND	NM_010507.1
*Ifna11*	Interferon alpha family gene 11	1.05	0.94	NM_008333
*Ifna12*	Interferon alpha family gene 12	ND	ND	NM_177361.2
*Ifna13*	Interferon alpha family gene 13	ND	ND	NM_177347.2
*Ifnar1*	Interferon (alpha and beta) receptor 1	1.87	4.84	NM_010508.1
*Ifnar2*	Interferon (alpha and beta) receptor 2	2.63	1.87	NM_010509.1
*Ifng*	Interferon gamma	ND	8.63	NM_008337.1
*Ifngr1*	Interferon gamma receptor 1	1.03	0.69	NM_010511.1
*Ifngr2*	Interferon gamma receptor 2	4.67	2.96	NM_008338.2
*Ifrd1*	Interferon-related developmental regulator 1	7.09	11.35	NM_013562
*Ifrd2*	Interferon-related developmental regulator 2	0.85	1.05	NM_025903.1
*Ifrg15*	Interferon alpha responsive gene	1.158	1.34	NM_022329.2
*Igtp*	Interferon gamma induced GTPase	1.92	2.57	NM_018738.2
*Irebf1*	Interferon response element binding factor 1	ND	9.45	NM_013714.1
*Irf1*	Interferon regulatory factor 1	5.43	1.96	NM_008390.1
*Irf2*	Interferon regulatory factor 2	1.01	0.93	NM_008391.2
*Irf2bp1*	Interferon regulatory factor 2 binding protein 1	0.68	0.82	NM_178757.3
*Irf3*	Interferon regulatory factor 3	1.24	0.93	NM_016849.2
*Irf4*	Interferon regulatory factor 4	2.63	1.73	NM_013674.1
*Irf5*	Interferon regulatory factor 5	1.81	1.57	NM_012057.1
*Irf6*	Interferon regulatory factor 6	1.11	1.02	NM_016851.1
*Irf7*	Interferon regulatory factor 7	1.60	3.18	NM_016850.1
*Isg20*	Interferon-stimulated protein	1.90	1.69	NM_020583.4
*Isgf3g*	Interferon dependent positive acting transcription factor 3 gamma.	2.27	1.24	NM_008394.2
*Jak1*	Janus kinase 1	1.30	1.25	NM_146145.1
*Jak2*	Janus kinase 2	2.26	1.44	NM_008413.1
*Mx1*	Myxovirus (influenza virus) resistance 1	8.02	16.69	NM_010846
*Oas1g*	2–5 oligoadenylate synthetase 1G	2.47	3.00	NM_011852.2
*Prkr*	Protein kinase interferon-inducible double stranded RNA dependent	1.69	2.63	NM_011163.2
*Prkrir*	Protein kinase interferon-inducible double stranded RNA dependent inhibitor repressor of (P58 repressor)	1.17	0.94	NM_028410.1
*Psmb8*	Proteosome (prosome macropain) subunit beta type 8 (large multifunctional protease 7)	1.41	1.54	NM_010724
*Ptpn2*	Protein tyrosine phosphatase non-receptor type 2	18.90	6.05	AK076072
*Socs1*	Suppressor of cytokine signaling 1	1.63	1.16	NM_009896
*Stat1*	Signal transducer and activator of transcription 1	1.34	1.25	NM_009283.2
*Stat2*	Signal transducer and activator of transcription 2	3.27	3.24	NM_019963.1
*Tap1*	Transporter 1 ATP-binding cassette sub-family B (MDR/TAP)	1.59	2.85	NM_013683.1
*Tyk2*	Tyrosine kinase 2	1.22	0.97	NM_018793.1

**Fold change**, lung interferon gene expression 2 or 4 h after NTHi lysate treatment, compared to untreated mice. **Accession**, GenBank accession number. **ND**, no gene expression detected at the designated time point.

### Prolonged protection without tachyphylaxis following repetitive dosing

**Figure 3:**
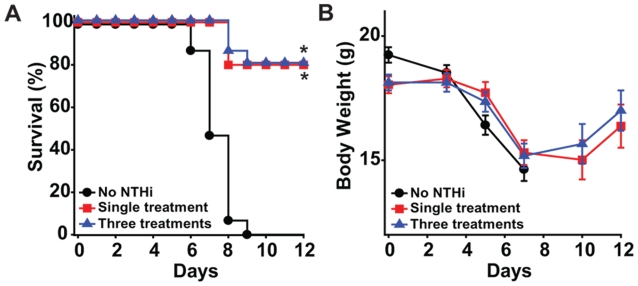
Repeated doses of aerosolized NTHi lysate provide prolonged protection against influenza virus without tachyphylaxis. (A) Mice were challenged with aerosolized influenza A without NTHi lysate pretreatment, following a single treatment on day −1 before infection, or following treatments on days −7, −4, and −1 prior to infection. Survival curves are shown (*p<0.0001). (B) Weight changes in the same groups of mice shown in A.

Since a high level of protection against influenza virus only lasts for 3 days (Fig. 1), prolonged protection would require repetitive dosing. To test whether tachyphylaxis to the protective effects of the aerosolized NTHi lysate occurs, we challenged mice with influenza in three different conditions: no lysate treatment, a single lysate treatment, or repetitive lysate treatment. We again observed 100% mortality in the untreated group, but found identical protection by NTHi lysate treatment whether given once one day prior to infection, or given three times (seven, four and one days prior to infection, Fig. 3A). In both NTHi lysate treated groups, protection was associated with recovery of early weight loss by the fifth day after infection (Fig. 3B). Observation for 21 days after the challenge showed no subsequent mortality.

### Treatment with a combination of aerosolized NTHi lysate and ribavirin after infection

**Figure 4:**
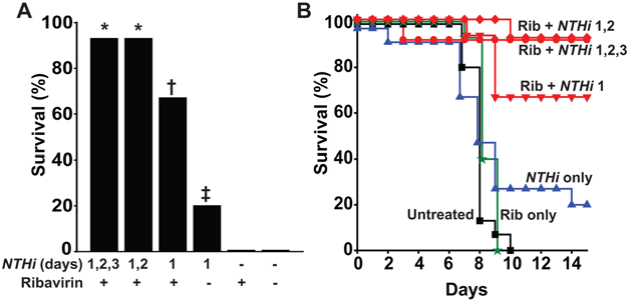
Aerosolized NTHi lysate combined with ribavirin effectively treats influenza virus pneumonia. After aerosolized challenge with ∼10^7^ TCID_50_/mouse influenza A, mice were treated with high dose ribavirin (Rib), NTHi lysate, or a combination of the two. Combination treated mice received ribavirin+NTHi therapy for the days indicated, up to three days. (A) Survival at day 14 for each of the conditions (*p<0.0001, †p = 0.0002, ‡p0.2 compared with untreated). (B) Survival curves for each of the conditions in A.

An alternative to repetitive stimulation of lung innate immunity for prophylaxis during an epidemic viral infection would be to treat with the pro-inflammatory aerosol after infection in combination with an antiviral drug. We recently found that treatment with high-dose aerosolized ribavirin after exposure to influenza virus provided some survival improvement in mice [19]. To test whether these two interventions might be effectively combined, mice were infected with influenza virus then treated with ribavirin alone, NTHi lysate alone, or a combination of the two. Ribavirin alone did not improve survival with the high level infectious challenge used in this study. However, as shown in Figure 4, suspending ribavirin in a single dose of NTHi lysate on day 1 after infection improved survival significantly more than NTHi lysate alone (66.7% vs 20.0% survival, p = 0.013), and an additional NTHi lysate treatment on day 2 almost completely protected from mortality (93.3% survival, p<0.0001 vs. control). No untoward effects or additional protection were noted if a third NTHi lysate dose was added on day 3. Observations through day 21 showed no subsequent mortality.

## Discussion

Lower respiratory tract infections are the leading cause of infectious death worldwide[1]. We have recently shown that Stimulated Innate Resistance (StIR) of the lungs induced by an aerosolized bacterial lysate can protect mice against otherwise fatal bacterial pneumonias [16]. We show here that the same treatment can prevent viral pneumonia, despite induction of lung inflammation of greater magnitude than that observed with lethal infection.

Influenza-induced excessive and/or dysregulated lung inflammation has been recently described as a mechanism by which pandemic infections cause mortality [10], [11], [12], [13]. Consequent to these observations, considerable effort has been invested in attempts to improve outcomes of influenza-infected mice through genetic and pharmacologic suppression of inflammatory cytokine production [12], [20], [21], [22]. In contrast, our current and previous data demonstrate robust inflammation associated with the induction of protective host responses (Fig. 3a–d). As we have previously shown that repetitive treatments result in diminishing inflammatory responses without loss of protection, it is possible that the cytokines themselves are not responsible for the protective effect. However, like our previous observations with protection against bacterial pathogens [16], the pro-inflammatory pretreatment resulted in a significant reduction in pathogen burden, here represented by decreased viral titers (Fig. 1d). As interferon signaling is associated with survival of viral and bacterial infections, the observation of augmented type I and II interferon signaling after NTHi lysate treatment (Figure 3A–B, Table 1) supports our hypothesis that this is also an important element of anti-viral StIR, and we will explore this in the future.

One manner in which the host response to NTHi lysate differs from reported pandemic virus-induced inflammation [11] is in its restriction to the lung. While documenting massive increases in indicators of lung inflammation following treatment, there is virtually no systemic inflammation noted (Fig 3c). Similarly, treatment with NTHi lysate induces no systemic leukocytosis (data not shown), despite the profound cellular infiltration induced in the lungs. We presume this confinement of the response to the lungs explains the lack of morbidity we have observed when mice are treated acutely even with very high doses of NTHi lysate [16]. Based on these observations, we conclude that influenza pneumonia does not kill via excessive pulmonary inflammation, but progresses through a deficit of effective inflammation.

In addition to exuberant lung inflammation, secondary bacterial infections are often considered important contributors to influenza-related mortality [13], [14], [15]. Interestingly, we here demonstrate that the host response to bacterial products actually enhances the clearance of a viral pathogen (Fig. 1D), just as we have shown for bacteria[16]. So, while viral infections may impair antibacterial interferon-γ responses [15], we observe no evidence that the response to the bacterial lysate impedes either type I or type II interferon signaling. Rather, the response to the bacterial products seems to reinforce protective antiviral events via vigorous interferon gene expression.

Epidemic respiratory infections, as previously observed with influenza [10], [11] and SARS [23], result in high mortality, sometimes before the pathogen is identified, and often without effective post-exposure treatment options. Seasonal influenza, though the case-mortality rate is lower, still kills ten of thousands of Americans annually [4], [5] and clinicians are faced with the problems of ineffective vaccine strategies and declining effectiveness of neuraminidase inhibitors [24], [25]. In such situations, rapidly dispersed, broad protection would be highly advantageous.

At present, the primary means to contain the spread of influenza and to prevent exposed individuals from developing disease is through the use of the trivalent hemagglutinin subunit vaccine or the live-attenuated trivalent vaccine coordinated by the United Stated Centers for Disease Control and Prevention. This strategy is limited by the fact that prevalent (potentially pandemic) strains may not be accurately predicted for inclusion in the annual vaccine, that normal adaptive immunity is required for protection against included strains, and that development of immunity takes weeks – potentially obviating the benefit in the setting of a rapidly spreading outbreak.

In this study, we showed that a single treatment with NTHi lysate was sufficient to prevent otherwise fatal influenza pneumonia (Fig. 1). The aerosolized lysate was also administered repetitively to model the sustained protection that would be clinically desirable during an influenza epidemic. No tachyphylaxis to the influenza-protective effect was observed with successive administrations (Fig. 2), despite the fact that the rise in lung lavage neutrophils induced by the aerosolized lysate becomes less with repeated administration [26]. These findings are consistent with the development of tolerance to the inflammatory effects of innate immune stimulation without loss of antimicrobial effector function, as recently described [27]. Alternatively, the progressive reduction in inflammation could reflect the tempering of innate immune responses by adaptive immune cells that become engaged with repetitive stimulation [28].

In addition to the preventive benefits of NTHi lysate-induced StIR, we show that stimulation of lung innate immunity can be therapeutically combined with an antiviral medication after influenza infection to improve survival more than either treatment alone (Fig. 4). In our current and prior work, we have reported only a modest survival benefit when the lysate is delivered 24 h after infection. However, we found that continuing induction of an antimicrobial inflammatory lung environment for up to 72 h potentiates the effects of high dose ribavirin and almost completely averts an otherwise uniformly fatal outcome. While Zheng and colleagues [22] recently reported improved influenza survival from 0% to 53.3% with the anti-viral/anti-inflammatory combination of zanamivir, celecoxib and mesalazine, we believe our current study to be the first to demonstrate such dramatic protection (improved from 0% to 93.3% survival) with an anti-viral combination known to induce inflammation. The seeming contradiction of these results may best be resolved by the hypothesis that the anti-inflammatory regimen diminished some toxicity associated with uncontrolled immune responses (i.e., reduced harmful inflammation) while our pro-inflammatory combination promoted the robust generation of defense effectors (i.e., enhanced protective inflammation).

In summary, we have shown that stimulation of lung mucosal innate immunity with a complex bacterial lysate confers striking protection against a virulent viral pathogen. Resistance to influenza is stimulated despite profound induction of lung-restricted inflammation, suggesting that excessive pulmonary inflammation is not the primary cause of influenza-induced mortality. Further, we found that the same treatment can be combined in the post-exposure setting with antiviral medication to improve survival. Taken together, we infer that therapeutic manipulation of StIR may be possible to reduce the mortality burden associated with viral pneumonias, and potentially, to protect the population against bioterror attacks.

## Methods

### Animals

Six to eight week old NIH Swiss-Webster mice (Charles River) were used for viral challenges. For protection studies, mice were divided into groups of 20 mice (5 for virus lung titers, 15 for survival). For bronchoalveolar lavage assays, an additional 5 mice were added for each group. Six to eight week old C57BL/6 mice were used for gene expression analysis, 6 mice per condition. Mice were handled in accordance with protocols approved by the Institutional Animal Care and Use Committees of Baylor College of Medicine and The University of Texas-M.D. Anderson Cancer Center, and euthanized if distressed.

### Bacterial lysate aerosol treatment

Non-typeable *Haemophilus influenzae* (NTHi) was stored, grown and harvested as described [16], [26]. The cell pellet was washed and resuspended in 20 ml 0.9% sodium chloride solution. This suspension was passed three times through an EmulsiFlex C5 cell disruptor (Avestin) at greater then 10,000 psi, then diluted to 4–5 mg/ml in 0.9% sodium chloride solution by bicinchoninic assay (Pierce) and centrifuged at 15,000×g for 10 min. The supernatant was collected, the protein concentration was adjusted to 2.5 mg/ml, and the lysate was sterilized by passage through a 0.2 µm filter and frozen in 8 ml aliquots at −80°C. Treatment of mice with aerosolized lysate was performed as described [16], delivering 8 mL of suspension during each 30 min exposure.

### Influenza A challenge

A clinical isolate of influenza A/Hong Kong/8/68 (H3N2) (A/HK; Mouse Lung Pool 11-29-05) virus that had been passaged at least nine times through mice was stored as frozen stock (2.8×10^7^ TCID_50_/ml) in the supernatant of mouse lung homogenates [29]. Stock was diluted 1∶300–1∶1,000 in 0.05% gelatin in Eagle's minimal essential medium (Sigma-Aldrich) and aerosolized for 20 min to achieve LD_90_–LD_100_ (target 100 TDIC_50_/mouse). Viral concentration in the nebulizer before and after aerosolization and in lung homogenates was determined by hemagglutination assay of infected MDCK cells [30]. In some experiments, 1 g of ribavirin was dissolved in 10 ml of NTHi lysate prior to aerosolization. Final ribavirin and lysate protein concentrations were 100 mg/ml and 2.5 mg/ml, respectively. Mice were challenged without pretreatment, following pretreatment on day −1, or following pretreatment on days −7, −4 and −1. On day 0, all groups of mice were exposed to the same viral aerosol. On day +4, 5 mice from each group were sacrificed and their lungs removed. Lungs were homogenized by beadbeating and the levels of virus determined. Remaining mice in each group were observed daily for up to 21 days for overt illness, morbidity and mortality. Mice were weighed on days 0 and +4, and three times weekly from day +7 until day +21.

### Host response to pathogen challenge

To characterize the inflammatory host response to treatment and/or infection lung lavage and cell counts were performed as described [16]. In order to assess the response to NTHi lysate treatment, lavage samples from unchallenged mice were obtained 4 h after the final (or only) NTHi lysate treatment. In order to collect samples during acute virus-induced illness, lavage samples were also collected on day +3 following infection with influenza. Multiplex ELISA cytokine analysis was performed by Searchlight Protein Array Analysis (Pierce Biotechnology). Total leukocyte count was determined with a hemacytometer, and differential count by cytocentrifugation of 300 µl of bronchoalveolar lavage fluid at 450×g for 5 min, followed by Wright-Giemsa staining.

### Gene expression analysis

To better understand the gene expression response to the treatment, mice were treated with the aerosolized NTHi lysate, then euthanized after 2 h or 4 h for comparison to untreated mice. To reduce the lung leukocyte burden, the pulmonary vasculature was perfused and the airways lavaged with PBS. The lungs were mechanically homogenized, then total RNA was isolated from lung homogenates using the RNeasy system (Qiagen), and cRNA was synthesized and amplified from equal masses of total RNA using the Ilumina TotalPrep RNA amplification kit (Ambion). Amplified cRNA was hybridized and labeled on Sentrix Mouse-6 Expression BeadChips (Illumina), then scanned on a BeadStation 500 (Illumina). Primary microarray data were deposited at the NCBI Gene Expression Omnibus (http://www.ncbi.nlm.nih.gov/geo/) consistent with MIAME standards (GEO Accession GSE13740). Primary signal intensity was normalized between and within samples, and differentially expressed genes were identified based on signal change and inter-sample variation. Gene ontology analysis was performed using the NIAID Database for Annotation, Visualization and Integrated Discovery (DAVID) and the KEGG Database (GenomeNet). Differentially expressed genes were mapped to signaling pathways using Ingenuity Pathways Analysis 5.0 (Ingenuity Systems), and the pathway nodules were individually reviewed. C57BL/6 mice were used for the gene expression studies as they demonstrate similar NTHi lysate induced protection against all investigated pathogens, and because the genetically manipulated mice the authors plan to use to dissect the mechanisms of StIR are primarily on C57BL/6 backgrounds.

To characterize the interferon-related gene expression changes induced by NTHi, Table 1 presents a list of genes containing all transcripts from the Ingenuity Pathway Analysis canonical interferon signaling pathway, all detected interferon-related JAK-STAT transcripts in KEGG, and additional interferon related transcripts identified by the authors. Baseline signal intensity values of 1 were assigned to undetected control transcripts in order to avoid reporting infinite fold change values.

### Statistical methods

Summary statistics for virus in lung tissue were compared using Student's t-test. Proportions of mice surviving pathogen challenges were compared using Fisher's exact text, and log-rank comparisons of survival distribution were performed using Kaplan-Meier estimation. All data shown are representative of at least two independent experiments, and were not combined for analysis because of modest differences in virus challenge doses. Analyses were performed using SAS/STAT (SAS Institute).

For gene expression analyses, treated and untreated samples were compared to identify treatment-induced gene expression using an ANOVA-based scheme written in R (Free Software Foundation, Boston, MA), utilizing an Illumina library developed by Simon Lin, Northwestern University, Chicago, IL.
